# Forest adjacent households’ voices on their perceptions and adaptation strategies to climate change in Kilombero District, Tanzania

**DOI:** 10.1186/s40064-016-2484-y

**Published:** 2016-06-21

**Authors:** Chelestino Balama, Suzana Augustino, Siri Eriksen, Fortunatus B. S. Makonda

**Affiliations:** Department of Wood Utilization, Sokoine University of Agriculture, P.O. Box 3014, Chuo Kikuu, Morogoro, Tanzania; Noragric, Department of Environment and Development Studies, Norwegian University of Life Sciences, P.O. Box 5003, 1432 Ås, Norway; Tanzania Forestry Research Institute, P.O. Box 1854, Morogoro, Tanzania

**Keywords:** Climate change, Perceptions, Forest adjacent households, Coping and adaptation strategies, Non-timber forest products, Tanzania

## Abstract

Climate change is a global and local challenge to both sustainable livelihoods and economic development. Tanzania as other countries of the world has been affected. Several studies have been conducted on farmers’ perceptions and adaptation to climate change in the country, but little attention has been devoted to forest adjacent households in humid areas. This study assessed this gap through assessing forest adjacent households’ voices on perceptions and adaptation strategies to climate change in Kilombero District, Tanzania. Data collection involved key informant interviews, focus group discussions and household questionnaires. Results showed that the majority of households perceived changed climate in terms of temperature increase, unpredictable rainfall, frequent occurrence of floods, increased dry spells during rainy season coupled with decreased water sources and emergence of new pests and diseases. The perceived change in climate has impacted agriculture productivity as the main livelihood source. Different coping and adaptation strategies are employed. These are; crop diversification, changing cropping calendar, adopting modern farming technologies, and increasing reliance on non-timber forest products. These strategies were positively and significantly influenced by socio-economic factors including household size, residence period, land ownership and household income. The study concludes that, there are changes in climatic conditions; and to respond to these climatic changes, forest adjacent households have developed numerous coping and adaptation strategies, which were positively and significantly influenced by some socio-economic factors. The study calls for actual implementation of local climate change policies and strategies in order to enhance adaptive capacity at household level.

## Background

Climate change is among the key challenges that hinder sustainable livelihoods and economic development, particularly for developing countries like Tanzania. The main livelihood source of the people is rain-fed agriculture, with only 2 % of arable land having irrigation facilities (Eriksen et al. 2005; Shemsanga et al. 2010; Ahmed et al. 2011). According to the Intergovernmental Panel on Climate Change (IPCC) (2014) an additional of about 1–3 °C of global temperature is expected by 2100 accompanied with variations in rainfall by regions. The National Adaptation Programme of Tanzania (NAPA) predicted the mean daily temperature to rise by 3–5 °C throughout the country and the mean annual temperature by 2–4 °C (URT 2007). The report further predicts that rainfall in some parts of the country will increase while others experience decrease (URT 2007). According to URT (2012), the change in temperature and precipitation patterns have led to increased risk of recurrent droughts and devastating floods, threats to biodiversity, an expansion of plant and animal diseases and a number of potential challenges on public health. People with low adaptive capacity are thought to be more vulnerable to the adverse effects of climate change, which contributes to the loss of their natural resources (Eriksen et al. 2005; Paavola 2008). Furthermore, Bakengesa et al. (2011) in Kilombero Ramsar Site revealed that change in rainfall patterns and inflow have affected habitats and dependent wildlife.

According to Van den Ban and Hawkins (2000), perception has been defined as the process by which information or stimuli is received from our environment and transformed into psychological awareness. Perception is important in climate change because it is one of the elements that influence adaptation process. The forest adjacent households’ ability to perceive effects of climate change is a key precondition for their choice to adapt (Maddison 2006, 2007; Gbetibouo 2009). Adaptation to climate change requires that forest adjacent households first realize that the climate has changed, and then identify useful adaptations and implement them (Maddison 2006, 2007). A better understanding of forest adjacent households’ perceptions of climate change, on-going adaptation measures, and the decision-making process is important to inform policies aimed at promoting sustainable adaptation strategies for all sectors of the economy. For example, currently in most areas, forest adjacent households perceive that the climate has become hotter and the rains are less predictable and shorter in duration (URT 2012). The change in rainfall and temperature trends and pattern (URT 2007, 2012) is expected to pose adverse effects on livelihoods of the people in most parts of the country. According to IPCC (2014) climate change adaptation is “a process of adjustment to actual or expected climate and its effects, in order to moderate harm or exploit beneficial opportunities”. According to Moser (2010) adaptation strategies can range from short-term to long-term, aim to meet more than climate change goals alone, and may or may not succeed in moderating harm or exploiting beneficial opportunities. Coping can be distinguished from adaptation as it refers to the use of existing resources to achieve desired goals during and immediately after climate-induced hazards (Regmi et al. 2010). In other words, coping is a short term strategy that is applied immediately after events in order to moderate harm. In a long run, some coping strategies may then be developed to become adaptation strategies. Reuveny (2007) urged that, individuals adapt to climate change in three main ways: stay in a place and do nothing, accepting the costs; stay in the place and mitigate changes; or leave affected areas. A significant majority of the forest adjacent households sustain their livelihoods by direct use of of forest resources including NTFPs. They also generate cash income from the trade of these products. These products are utilized by forest adjacent households to meet subsistence needs and currently act as economic base and safety nets during periods of climate induced stresses (Paavola 2008; Nkem et al. 2010). Sustainable utilization of forest products especially NTFPs can significantly contribute to increasing the adaptive capacity of the forest adjacent households through improved food and health security, increased well-being and increased cash income.

In this regard, frequency and intensity of extreme weather events such as drought and floods have increased in some parts of Tanzania affecting climate sensitive sectors including agriculture (URT 2008). A number of climate change perceptions and adaptation studies conducted in Tanzania (Naess 2008; Lema and Majule 2009; Nelson and Stathers 2009; Lyimo and Kangalawe 2010; Mongi et al. 2010; Swai et al. 2012; Kangalawe and Lyimo 2013; Kihupi et al. 2015) focused on smallholder farmers in semi-arid areas with little attention to humid areas and specifically the forest adjacent households whom the majority have varied livelihood activities. This study assessed forest adjacent household’s voice on their perceptions and developed adaptation strategies against climate change effects in Kilombero District, Tanzania. The study addressed the following specific questions: (1) How do the forest adjacent households perceive climate change and its effects in line with existing empirical data? (2) How are the forest adjacent households cope and adapt to the effects of climate change? (3) What socio-economic factors influence their coping and adaptation strategies? The findings have generated empirical information on forest adjacent households’ perception on climate change that can inform different national existing strategies and policies for example the climate change strategy and NAPA. Furthermore, results are useful to policy and decision makers within the natural resource sectors in order to implement existing policies (National Agriculture and Forest policies) and strategies [reduced emission from deforestation and forest degradation (REDD+) and the National Climate Change] through developing and strengthening the existing coping and adaptation strategies.

## Methods

### Description of the study area

**Fig. 1:**
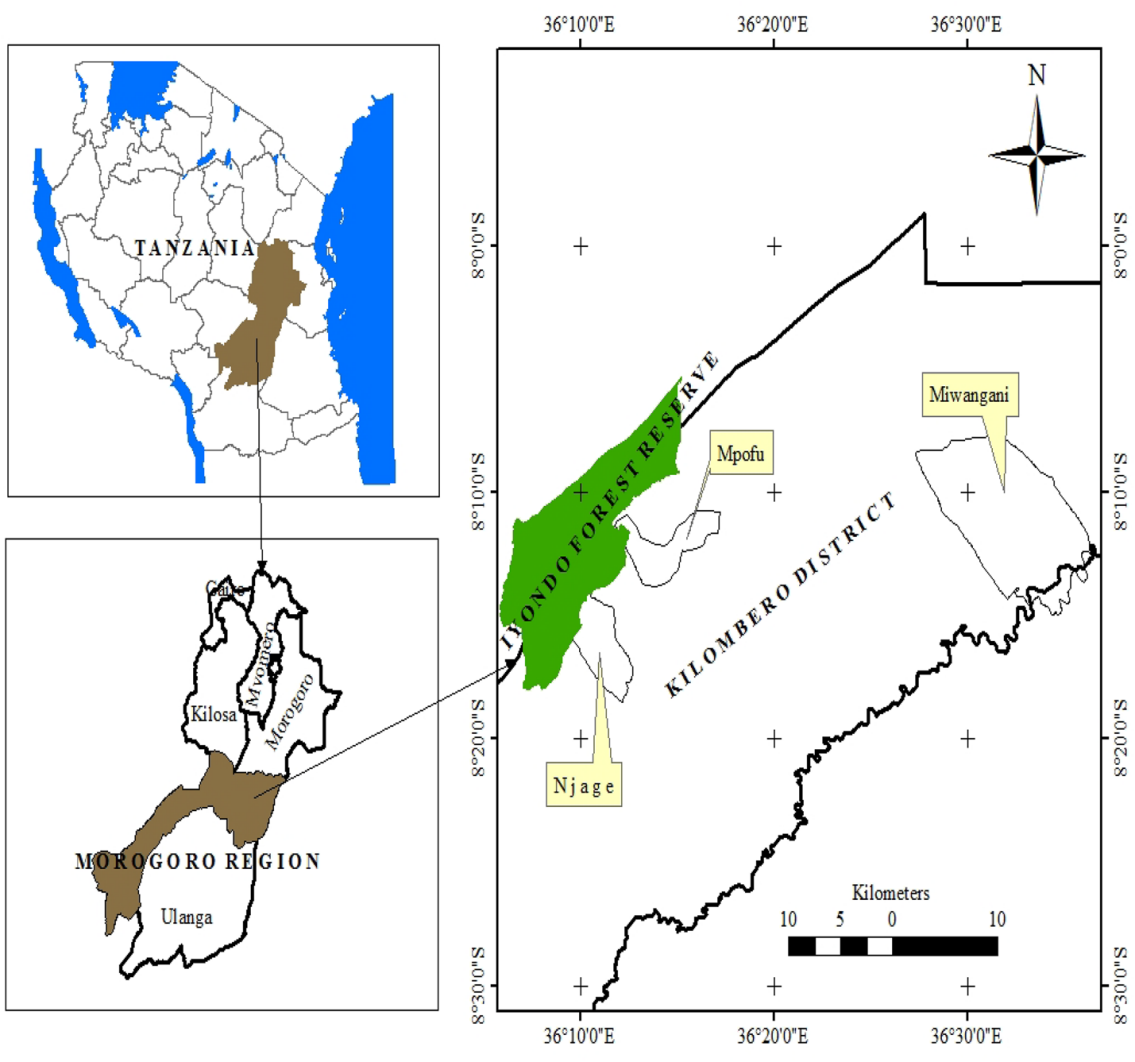
A map showing location of study villages in Kilombero District, Tanzania

Kilombero District is among the six districts of Morogoro Region (URT 2013a). A large part of Kilombero District is located in a floodplain area, which is important for main economic activities of the people including agriculture, livestock keeping, fishing and wild game hunting (Kato 2007). However, these livelihood activities have been affected by the recurrent stresses due to changing climatic conditions including floods, dry spells during rainy season, extreme heat and some other stresses, which have adverse effects to the livelihoods of the people (Chamwali 2000; Starkey et al. 2002; Harrison 2006). Specifically the study was conducted in three villages of Kilombero District namely Mpofu, Njage and Miwangani (Fig. 1). The choice of the study area was based on villages that were highly affected by the changing climatic conditions and in the same time adjacent to Iyondo Forest Reserve (IFR) and wooded grassland (Lovett and Pocs 1993; Harrison 2006) with resources important for increasing resilience of the forest adjacent households against adverse effects of climate change.

The climate in the study area is marked by wet and dry seasons which are further distinguished into four sub seasons namely; hot wet season from December to March, cool wet season April–June, cool dry season July–August, hot dry season September–November. The area receives between 1200 and 1800 mm of rainfall per year and temperatures ranging from 26 to 32 °C (Erlanger et al. 2004; Hetzel et al. 2008). However, the rains are currently unpredictable whereby, the onset and cessation is inconsistent. The general topography in the study villages is a flat land with mean elevation ranging from 262 to 358 m a.s.l. Soils are mainly loamy and sandy while some cotton black soil in flooded areas are found. In hilly areas, the soils are sandy loam over crystalline rocks (Lovett and Pocs 1993).

Access to the study villages is through an earth road running from Ifakara Town to Mlimba as well as the railway line of Tanzania–Zambia Railway Authority (TAZARA) which extends from Dar es Salaam (Tanzania) to Kapiri Mposhi (Zambia). The main vegetation found in the study area is Miombo woodlands (Lovett and Pocs 1993), with some open grassland areas in the floodplains (Kato 2007; Laswai 2011). The study area is estimated to have more than 50 ethnic groups, but they still share similar livelihoods and socio-cultural norms due to type of available natural resources. The most dominant ethnic groups are Pogolo, Hehe, Ndamba, Bena, Ndali and Sukuma. Specifically, the Hehe ethnic group dominates in both Mpofu and Njage villages. This is due to migration of people from Kilolo District to Kilombero District because of reported better weather conditions for agriculture compared to places of their origin. The main economic activities in the study area are agriculture and livestock keeping. The main food crops grown include rice, maize, banana, cassava, fruits and horticultural crops (Kato 2007). Table 1 indicates some physical and demographic characteristics of the study area.

**Table 1:** Some characteristics of Mpofu, Njage and Miwangani villages in the study area

Characteristics	Mpofu	Njage	Miwangani
Geographical position	08°12′57″S; 36°14′333″E	08°15′26″S; 36°10′08″E	08°11′06″S; 36°32′11″E
Mean altitude (m)	295	312	272
Population^a^	3123	3402	2545
Average household size	4.72	4.78	4.93
Average land size (ha)/household	1.32	1.30	1.77
Crops grown	Main (banana, maize, rice); others (sesame, cocoa, sunflower, cassava)	Main (rice, banana, maize); others (sesame, cassava)	Main (rice, maize); others (sesame, cassava, banana)
Ethnic groups	Main (hehe, nyakyusa, bena, ndali); others (sukuma, makua, kerewe, gita, ndamba, pogolo, nyamwezi, haya, safa and kinga)	Main (hehe, bena, nyakyusa, ndamba); others (sukuma, pogolo, kerewe, gita, nyamwezi, chaga and matumbi)	Main (ndamba, pogolo, hehe, and sukuma); others (nyakyusa, ndali, luguru, bena, kinga and matumbi)

^a^Housing and population census of 2012 (URT 2013b)

### Data collection methods

Different methods and techniques were employed to collect qualitative and quantitative data from both primary and secondary sources. Primary data sources included participatory assessment and households’ survey. Participatory assessment methods included focus group discussions (FGDs) and key informant interviews. In each village, one FGD was held which comprised of 10–12 people of ≥40 years selected at random (in order to acquire climate information for the past 30 years) at the same time representing various livelihoods and gender. The key informants were drawn from District Agricultural and Forest officers, village leaders, Village Agricultural Extension officers and elderly people in the respective villages. The tools aimed at capturing information on the forest adjacent households’ perception on climate change; trend of rainfall and temperature, change in crop types and cropping pattern.

Household survey was conducted using structured questionnaire to complement the qualitative information from participatory assessment. Household was a sampling unit of analysis, because it is where all decisions about production, investment and consumption are primarily taken (Thomson and Metz 1998). A village register was used to select the households through assigning random numbers to each household. Then random selection was done from a list of random numbers to select sample households for questionnaire survey. A total of 215 households were randomly selected based on sampling intensity of 10 % (Table 2). Pilot testing of the survey instruments was conducted to among 30 randomly selected households prior to implementation of the survey and then the questions were adjusted accordingly. Pilot testing was carried out in order to improve validity of the survey tools (Barribeau et al. 2015). The household questionnaire was used to collect data related to; demography, forest adjacent households’ perceptions and responses to the adverse climate change effects as well as the socio-economic factors influencing climate change adaptation strategies in the study area.

**Table 2:** Sample size distribution in the study villages in Kilombero District, Tanzania

Village	Number of households	Sampling intensity	Sample size (n)
Mpofu	714	.1	70
Njage	868	.1	87
Miwangani	581	.1	58
Total	2163		215

Secondary data both published and unpublished literature from various sources was used to supplement primary data. Climatic data mainly rainfall and temperature from the Tanzania Meteorological Agency (TMA) for a span of 30 (1980–2010), and 20 (1990–2010) years, respectively were also used to supplement forest adjacent households perceptions on climate change in the study area.

### Data analysis

Content method and Statistical Package for Social Science (SPSS) were used to analyse qualitative and quantitative data respectively. Qualitative data collected through FGDs and key informant interviews were categorized into meaningful units and themes for triangulation with the quantitative information. Quantitative data mainly demographic characteristics, forest adjacent households’ perceptions to climate change effects and adaptation measures were coded, processed and analysed using SPSS computer software. Descriptive and multinomial logistic regression statistics were used in data analysis. Descriptive statistics generated percentage and means for variations on different variables in the study area. Multinomial logistic regression analysis was used to analyse the socio-economic factors influencing adoption of developed climate change coping and adaptation strategies. The multinomial logistic regression is an analytical approach that is commonly used in adoption decision studies involving more than two multiple choices (Greene 2002). In this case there were three categories: households without adoption strategies; households with one up to three strategies and lastly households with more than three strategies. The approach is also appropriate for evaluating alternative combinations of adaptation strategies, including individual strategies (Wu and Babcock 1998). The following multinomial logistic regression equations were used:

1 $${\text{Log}}\,[{\text{P}}({\text{adoption}}\,1{{-}}3\,{\text{strategies}}/(1 - {\text{P}}({\text{no}}{\text{-adoption}})))] =\upbeta_{0} + \sum\upbeta_{\text{i}} {\text{X}}_{\text{i}} +\upvarepsilon_{1}$$

2 $${\text{Log}}\,[{\text{P}}({\text{adoption}} > 3\,{\text{strategies}}/(1 - {\text{P}}({\text{no}} {\text{-adoption}})))] =\upbeta_{0} + \sum\upbeta_{\text{i}} {\text{X}}_{\text{i}} +\upvarepsilon_{1}$$

where P = probability function that a household adopts 1–3 strategies or more than three strategies, (1 − P) = is the probability that a household does not adopt any strategies, β_0_ = constant term of the model without the independent variables, β_s_ = are parameter estimates for the independent variable, X, ε = is an error term which represents unobservable factors assumed to be independently distributed over the survey period, X = is a vector of socio-economic factors, which include.

- Age (X_1_)—this is a continuous explanatory variable measured from the age of respondent. Studies show presence of relationship between age of household head and adaptation (Dolisca et al. 2006; Tazeze et al. 2012).
- Household size (X_2_)—this is a continuous explanatory variable that was measured from number of members of the respondents’ household.
- Education level (X_3_)—is a dummy explanatory variable which was given 1 to denote respondents with formal education (primary, secondary, college and university) and 0 for otherwise. Educated and experienced head of households are expected to have more knowledge and information about climate change and agronomic practices that they can use in response.
- Residence period (X_4_)—this is a continuous explanatory variable that was measured from number of years the respondent has lived in the study area. Duration a household head spent in the area for living was related to increased experience about the area. This included gained knowledge and information about agronomic practices and climate change.
- Land ownership (X_5_)—is a binary explanatory variable that was given 1, when respondent declared to own land and 0 for otherwise.
- Household income (X_6_)—this is a continuous explanatory variable that was measured from the annual total household income. The income was mainly from agriculture, formal employment, petty business, casual labour, sales of non-timber forest products (NTFPs), livestock and remittances.
- Forest access rules (X_7_)—this is a binary explanatory variable that was given 1, when respondent declared that rules and regulations enable access to forest products and 0 for otherwise.

## Results and discussion

### Forest adjacent households’ perception on climate change

The majority of forest adjacent households perceived the climate of their area to have changed. This is indicated by increase in amount of rainfall (68 %), delay in onset of rain (82 %) with high intensity and for a short time of raining (62 %) and temperature increase (75 %) in the area (Table 3).

**Table 3:** Forest adjacent households’ perception on climate change in Kilombero District, Tanzania

Indicator	Attributes	Response (%) n = 215
Rainfall amount	Increase	68
	Decrease	27
	No change	5
Rain season^a^	Delay in onset	82
	Early cessation	35
Rainfall intensity	High rains for a short time	62
	Little rains for a long time	23
	Little rains for a short time	11
	No change	4
Temperature	Increased	75
	Decreased	19
	No changed	6

^a^Multiple response analysis was applied to rain season variable

#### Rainfall amount and intensity

FGD participants noted that in the past (1970–1990s) the rainy season started in October, but currently rains are not as predictable as before. They also noted that rains sometimes start either in mid-December or early January, a situation that has forced them to change cropping calendar. One of the respondents in Njage Village during questionnaire survey explained more that as quoted:

- …as currently rains are starting late, we sow before tilling so that when rains set they are already in the soil, unlike to the past (1980–1990s) where we tilled the land first before sowing. This was also a way of reducing weeds because they grow fast soon after the onset of rains. This cropping system is not good for us because it increases the costs for weeding as we sow seeds before weeds have come out.

Similarly, the first rain season cessation was mentioned to be earlier than it was in the past two decades (1980–1990s). These two perceptions were consistent with the perceived shorter rainy season in all study sites. The shorter rain season resulted in reduction of crop yields because some of the crops failed to mature. The current findings are in line with those of Urama and Ozor (2011) in the Western and Central Africa where they revealed a decrease in length of the growing season and yield potentials due to climate change and called for advocated agricultural innovations for adaptation.

Climatic hazards such as increasing amount and intensity of rainfall was reported to cause floods on agricultural fields, impacting the livelihoods of forest adjacent households (Table 4) and mostly the Miwangani village households who are situated at lower elevation compared to Mpofu and Njage villages. Houses and roads were also reported to be destroyed by floods in all villages hence increasing the number of homeless households and disconnecting communication especially access to social services such as hospitals and schools.

**Table 4:** Effects of changing climatic conditions to forest adjacent households’ livelihoods in Kilombero District, Tanzania

Climate change effect	Responses (%) n = 215
High risk of floods on agricultural fields	99
Wilting of crops due to moisture stress	77
Settlements destructions due to floods	14
Increased outbreak of human diseases	24
Increased outbreak of pests and diseases in crop and livestock	18
Drying of rivers and dams	13
Increased wild fires on forests and grasslands in prolonged dry season	3
Roads destructions due to floods	4

#### Increased temperature

Increased temperature was reported to cause moisture stress in crops as well as increase prevalence of both animal and plant pests and diseases. The majority (79 %) of the respondents reported that crops have been wilting due to moisture stress, thus reducing crop yields. Moisture stress has also been related to an outbreak of army worms (pests) and rice yellow mottle virus (RYMV) disease in rice crops. These results concur with observations made by Akponikpe et al. (2010) in West Africa and Sanga et al. (2013) in Pangani River Basin and Pemba in Tanzania, where both reported moisture stress as a fundamental cause of increasing susceptibility of the crops to other stresses like pests and diseases, leading to reduced crop yield. According to Allarangaye et al. (2006) and Michel et al. (2008), RYMV is the most important virus disease for rice in Africa which reduces paddy productivity.

Household interview also revealed that increased temperature was also related to increased dry spells during rainy season(s). This was also the main cause for drying of rivers, streams and dams, causing water shortages to forest adjacent households (Table 4). The shortage of water at household level was claimed to be the source of outbreak of human diseases such as diarrhoea, typhoid, dysentery and amoebiasis. It was also noted that, cholera occurred mostly during periods of floods due to increased rate of bacteria that cause the disease. Similar observations have been pointed out by Traerup et al. (2010) that, high rate spread of waterborne diseases may be boosted by extreme climate conditions that enable the disease vectors spread more easily. Other human diseases that were reported to be associated with increased temperature in the study area included malaria, skin rashes, tick borne diseases and diarrhoea.

**Fig. 2:**
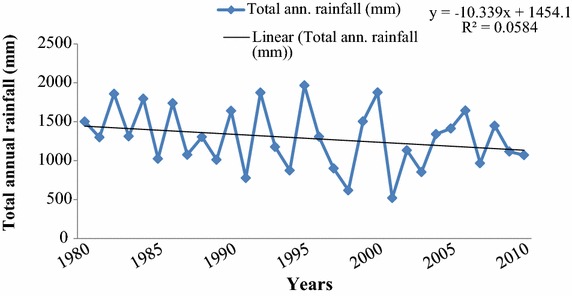
Total annual rainfall (mm) recorded between 1980 and 2010 in Kilombero District, Tanzania

**Fig. 3:**
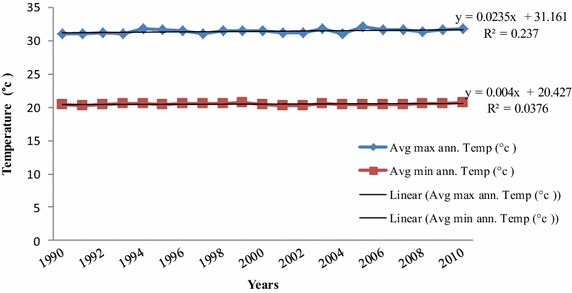
Average maximum and minimum temperature (°C) recorded between 1990 and 2010 in Kilombero District, Tanzania

The forest adjacent households’ perceptions on change in climate have been supported by existing empirical data from the TMA. The empirical climate data showed that rainfall and temperature in the study area have been unpredictable (Figs. 2, 3). The rainfall pattern from 1980 to 2010 showed a trend of decrease in total rainfall received. However, the trend was not significantly different at 5 % probability level. The empirical climate data corresponds to the perceptions of the forests adjacent households who reported recurrence dry spells in recent years. Furthermore, temperature trends have indicated a slight increase in both maximum and minimum average annual temperature, in the study area over the past decades, implying prolonged dry spells in the area.

#### Prolonged dry spells

FGDs participants revealed that prolonged dry spells especially in wet season increases chance for termites to attack crops, construction materials such as thatch grass, withies and ropes. This in turn increases frequency of collection of construction materials hence reducing overall productivity at household level particularly on food crops. Sileshi et al. (2009) indicated that some incidences of increase in termite attack on people’s assets have been associated with climate change-induced dry spells. Prolonged dry spells was also considered the major driver to increased wildfires which affect forests and grasslands. Wildfires were reported (Table 4) to be a serious threat in Njage village forest as well as in the grasslands in Miwangani and Mpofu village. For example, it was reported that Iyondo Forest Reserved has been much susceptible to wildfires especially in dry seasons (Table 4). Livestock (mainly cattle and poultry) were reported to be vulnerable to both prolonged dry spells and flooding, due to scarcity of pastures and feeds. Similar observations were noted by the IPCC Fourth Assessment Report (IPCC 2007) where extended warm periods and increased droughts led to increase in water stress in forests and grasslands and increased frequency and intensity of wildfires. According to Khandlhela and May (2006), poor quality of pastures due to floods and dry spells increased vulnerability of cattle and goats to pests and diseases, causing deaths in Limpopo Province, South Africa.

### Coping and adaptation strategies developed to respond to climate change effects

Following the realised adverse effects of climate change, forest adjacent households have acted in numerous ways. As Reuveny (2007) urged on how people respond to climate, in this study forest adjacent households were responding to climate change by staying in the place and mitigating the changes through various coping and adaptation strategies. Their responses based on the main adverse effects of climate change to their livelihoods, included coping and adaptation against dry spells, floods as well as pest and diseases.

#### Response to dry spells

During FGDs participants reported that, the government provided relief food to all forest adjacent households to cope with food shortage during dry spell periods. However, the supplied food was insufficient to carter for reasonable number of days that the forest adjacent households were in need. Apart from the government strategy of provision of relief food, the majority of forest adjacent households in the study area responded to the effects of dry spells that occurred during wet season through crop diversification, changing cropping calendar and adopting modern farming techniques (Table 5). Few (8 %) of the interviewed respondents said they did not have adaptation strategies. However, during FGDs it was revealed that, some of them practiced some coping/adaptation strategies unknowingly. This indicates that the respective forest adjacent households were involved in developing and practising adaptation/coping strategies. It was not easy for some respondents to link some agricultural practices with climate change adaptation.

**Table 5:** Developed forest adjacent communities strategies against dry spell effects in Kilombero District, Tanzania

Strategies	Responses (%) (n = 215)
Crop diversification	85
Changing cropping calendar	80
Adopting modern faming technologies	63
No adaption option	8

Crop diversification involved growing different varieties of food and cash crops, some of which are resistant to dry spells and some pests and diseases. Crop diversification aimed to supplement traditional crops such [*Oryza sativa* (L.)], banana (*Musa* spp.) and maize [*Zea mays* (L.)] as with non-traditional ones like sesame [*Sesamum indicum* (L.)], cassava (*Manihot esculenta* Crantz), sweet potatoes [*Ipomoea batatas* (L.)] and cocoa (*Theobroma cacao* (L.)] (Table 6).

**Table 6:** Main crops (traditional and non-traditional) grown in Kilombero District, Tanzania

Crops grown		Responses (%) (n = 215)
Common names	Botanical names	
Traditional crops		
Paddy	*Oryza sativa* (L.)	89
Maize	*Zea mays* (L.)	89
Banana	*Musa* spp.	52
Coconut	*Cocos nucifera* (L.)	1
Non-traditional crops		
Sesame	*Sesamum indicum* (L.)	20
Cassava	*Manihot esculenta* Crantz	22
Sweet potatoes	*Ipomoea batatas* (L.)	17
Cocoa	*Theobroma cacao* (L.)	3
Groundnuts	*Arachis hypogaea* (L.)	2
Pigeon peas	*Cajanus cajan* (L.)	13
Cow peas	*Vigna unguiculata* (L.) Walp	4
Sunflower	*Helianthus annuus* (L.)	3

During FGDs it was revealed that forest adjacent households have been growing fast growing food crop varieties including *Stuka* and *Staha* for maize and SARO 5, TXD 88 and TXD 85 for rice. Cash crops like sesame, cocoa and sunflower constituted recently introduced crops to diversify agricultural crops. In Mpofu Village for example, sesame has been grown since 2000, while in Njage and Miwangani it started in 2007. Sunflower crop is becoming famous in recent years in the study area because of its tolerance to moisture stress. Similar studies conducted elsewhere (Ellis 2000; URT 2007; Paavola 2008; Sanga et al. 2013) have indicated the importance of crop diversification as a way of adapting to the adverse effects of climate change.

Change in crop growing calendar was also reported by the majority during both FGDs and household interviews in the study villages (Table 5). The forest adjacent households were shifting crop growing calendar through early cultivation since rains were neither reliable nor predictable. Early planting was claimed to be a viable climate change adaptation mechanism as it allowed optimization of unpredictable, unreliable rainfall by ensuring crops were already established on the farm when the rains commence. It was noted that in the past decades (1970–1990s) forest adjacent households received short rains in October or November which continued to January. Currently, the short rains in November are used for seed sowing, compared to the past where it was time for weeds to germinate. This implies that, crops and weeds germinate at the same time, therefore increasing weeding costs, and sometimes reducing crop strength because of competing for nutrients and moisture.

With regard to adoption of modern farming, the study revealed availability of irrigation canal that was constructed by the Government of the United Republic of Tanzania in 2006 in Njage village to be used for rice production. The irrigation scheme was constructed in order to supplement water in some agricultural fields that were cultivated during both dry and wet seasons. In the 1970–1990s, such fields used natural spring water and some intermittent rains which currently are not available. Similar findings were recorded by Sanga et al. (2013) in Pangani River Basin and Pemba in Tanzania where farmers use irrigation system to adapt to dry spells.

#### Response to floods

During FGDs the forest adjacent households reported that, the government and some NGOs including the Plan International were involved in provision of camping facilities, food and health services to all affected people during occurrence of floods. Also the government was ahead in rehabilitating roads which were destroyed by the floods. The forest adjacent households claimed to respond to floods by increasing their reliance to casual labouring, use of NTFPs and petty trade (Table 7) for cash income generation than it used to be over the past decades. NTFPs like building poles were collected in order to re-establish houses destroyed by floods. Basically the use of NTFPs as part of coping to climate change was referred to when affected individuals collected these products for trade to get income to buy their immediate needs like food, medicines and construction materials. It was revealed that not all of the NTFPs were used for coping but some like firewood was already part of their adaptation strategies as is being practised throughout the year. It was note that income of individuals in the study area depends mostly on sales of agricultural produce, which become scarce during farming season when floods occur. When houses and other infrastructure are destroyed, the local income of the forest adjacent households becomes limited to few sources like sales of forest products. Generally, use of NTFPs for adaptation in most cases was rather indirect way particularly when one get cash income and use it for other immediate needs.

**Table 7:** Developed strategies for managing changing climatic conditions in Kilombero District, Tanzania

Coping strategy	Responses (%) (n = 215)
Casual labour	20.2
Use of NTFPs	17.3
Selling livestock	6.2
Remittance	2.8
Petty trade	5.6
Selling rice and buying maize	1.1

From Table 7, some (20 %) of the forest adjacent households were involved in casual labour activities for income generation in order to cope with the frequent floods that occurred. The use of NTFPs to cater for food, construction and income generation that was used for various domestic needs during flood related hardships was also mentioned by a small proportion (17 %) of respondents. The commonly mentioned NTFPs included mushrooms, firewood, medicinal plants and edible fruits which were illegally collected from IFR and village woodlands or collected from own farms. During FGDs forest adjacent households mentioned that, village woodlands and on farms were the most reliable alternative sources of NTFPs that could provide the products if sustainably managed. Results on NTFPs use for livelihood sustenance imply they could form part of the coping and adaptation strategies for forest adjacent households’ resources if well developed to ensure sustainability. Reliance on NTFPs was high for cash and non-cash income because main livelihood activity (agriculture) has adversely been affected by changing climatic conditions. Similar findings have been reported by Nkem et al. (2010) in the Democratic Republic of Congo (DRC) whereby loss of income of the people due to climate change have necessitated them to go into commercialization of NTFPs such as medicinal plants, mushroom, caterpillars, fish, bush meat and palm wine for adaptation. This could apply to the people in Kilombero District if domestication and value addition could be emphasized for the existing NTFPs in order to enhance forest adjacent households’ adaptive capacity and in turn help to improve income and food security at household level. Adaptation through use of NTFPs has also been proposed in the IPCC Firth Assessment Report (Smith et al. 2014) as important for the climate resilience of local livelihood systems because natural forests are more resilient to climate change effects than monoculture plantations.

The study has also revealed prevalence of a shift in use patterns of NTFPs from subsistence to trade for income generation (Table 8). NTFPs were both consumed and traded to get income that was used for various human needs. According to Nkem et al. (2010), “NTFPs are fundamentally the niche for poor population, which make them important for addressing poverty, health problems and adaptation to external shocks and stresses”. The current study recorded relatively higher annual average of collected firewood that was traded than that used for subsistence which was 292.8 ± 14.3 and 116.5 ± 4.6 head loads, respectively. Similarly, annual average consumption per household of medicinal plants and thatch grasses were also high (Table 8). This indicates that most of the forest adjacent households used NTFPs for trade in order to get immediate cash income that was used for various household needs including health services, food and shelter especially when main source of income (i.e. agriculture) is adversely affected by floods.

**Table 8:** Use pattern of priority NTFPs by households in Kilombero District, Tanzania

Use pattern	Priority NTFPs	Annual average collection per household
Subsistence	Firewood	116.53 ± 4.63 (77)
	Medicinal plants	5.53 ± 0.35 (47)
	Thatch grass	31.33 ± 1.71 (63)
Trade	Firewood	292.8 ± 14.27 (23)
	Medicinal plants	16.39 ± 2.72 (15)
	Thatch grass	40.06 ± 1.66 (54)

Unit for firewood and thatch grasses is head load, equivalent to 16.55 ± 3.33 and 14.12 ± 3.19 kg, respectively; while for medicinal plants is kg; numbers in parenthesis are respondent frequencies (%)

It was revealed that the use of NTFPs for climate change adaptation was gender sensitive with both involvement of men (54 %) and women (46 %) for commodities such as mushrooms (Table 9). In the past activities like mushroom and firewood collection were done by women only and men were mainly involved in game meat hunting, collection of building poles and timber harvesting.

**Table 9:** Types of NTFPs as adaptation to climate change effects by gender in Kilombero District, Tanzania

NTFPs	Responses (%) (n = 215)	
	Male	Female
Wild mushrooms	54	46
Construction materials (thatch grass, withies, ropes, poles)	12	13
Firewood	14	15
Edible fruits	3	3
Edible tubers	14	10
Medicinal plants	3	4
Malala [*H. compressa* (H) Wendel.]	53	27
Ukindu (*P. reclinata* Jacq.)	13	23

The difference in gender roles within NTFPs as adaptive measure among user groups in the study area could probably be attributed to socio-cultural factors within the gender roles as collection of firewood, wild vegetable and mushrooms in the past was mainly done by women. The findings in the current study concur with Msuya et al. (2010) in the Eastern Arc Mountains, Tanzania. Currently men in the study area have been forced by the climate change adverse effects to be involved in some activities they were not used in the past in order to earn cash income. However, there might be some other factors like increased demand (market forces) of the resources due to dwindling supply and increased population in towns and formation of new villages. Similar results have been reported by Augustino et al. (2012) and Msalilwa et al. (2013) in selected parts of Tanzania. The findings in this study are also similar to those recorded by Nindi and Mhando (2012) in Mbinga District, where people used mushrooms, medicinal plants and construction materials as their immediate coping strategies to climate change effects.

On the other hand, use of weaving materials locally known as malala [*Hyphaene compressa* (H.) Wendel.] to make various products was revealed to be a strategic activity by more men (53 %) compared to women (27 %) (Table 9). This was due to the fact that women preferred much another type of weaving material known as ukindu (*Phoenix reclinata* Jacq.) due to its high value for the final product compared to malala. The use of NTFPs as an adaptation strategy is well emphasized in NAPA especially during periods of extreme weather conditions (URT 2007). The study suggests the need to strengthen this type of adaptation to ensure sustainable livelihood of forest adjacent households in future.

#### Response to pest and diseases

During FGDs, forest adjacent households revealed the use of different plant species for treatment against some human diseases such as cholera, typhoid, dysentery and amoebiasis. Malaria, stomach ache, skin rushes, cold and cough, as well as some non-communicable diseases like diabetes and hypertension. Government intervention was also reported which included provision of mosquito nets as a protective measure of malaria. Also during outbreaks of cholera, the government took care of all affected people through establishment of quarantine centres. In the quarantine centres people were provided with all necessary treatment and preventive measures against cholera. Frequent outbreak of pests and diseases (most of them being due to adverse climate conditions) necessitated forest adjacent households to rely much on medicinal plants because most of them were not able to afford modern treatments because of either limited health centres or cash income. Most of the popular remedies were reported to be common to the majority of the forest adjacent households. Dependence on medicinal plants as a primary healthcare by rural people has also been reported elsewhere in Tanzania (Kitula 2007; Otieno et al. 2011; Kayombo et al. 2013; Njana et al. 2013; Nahashon 2013; Augustino et al. 2014). Nkem et al. (2010) urged that dependency on traditional medicine is perpetuated by the fact that artificial medicinal care is limited in rural areas, and where it is available the costs are relatively high.

### Socio-economic factors influencing climate change adaptation strategies by households

The results showed that, household size, residence period the respondent lived in the study area, land ownership and household income were the socio-economic variables that positively influenced significantly adaptation strategies (P ≥ 0.05) (Table 10). Age of the respondent has negatively influenced significantly adaptation strategies at (P ≥ 0.05). At the same time, education level influenced adaptation strategies positively but not significantly (P ≥ 0.05). Forest access rules influenced adaptation strategies negatively and insignificantly (P ≥ 0.05).

**Table 10:** Socio-economic factors influencing climate change adaptation strategies in Kilombero District

Independent variables	One–three adaptation strategies						More than three adaptation strategies					
	β	SE	Wald	t	Sig.	Exp(β)	Β	SE	Wald	t	Sig.	Exp(β)
Intercept	.490	.776	.399	1	.528	−	−20.921	4.939	17.940	1	.000	−
Age (X_1_)	−.038	.016	5.819	1	.016*	.963	−.045	.127	.124	1	.725	.956
Household size (X_2_)	.400	.081	8.569	1	.003*	1.269	.400	.332	1.451	1	.228	1.492
Residence period (X_3_)	.007	.012	6.815	1	.009*	1.031	.007	.097	.005	1	.941	.993
Education level (X_4_)	.464	3.059	1.235	1	.266	.629	16.251	3.059	.000	1	.998	.000
Land ownership (X_5_)	16.399	.000	1.023	1	.312	.575	16.399	.000	.000	1	.000*	1.325
Household income (X_6_)	.017	.042	3.093	1	.049*	1.031	.067	.097	.005	1	.941	.993
Forest access rules (X_7_)	−.002	.385	.000	1	.996	.998	−1.655	7.261	.000	1	.998	1.5397

Cox and Snell R^2^ = .261, Nagelkerke R^2^ = .342, β = regression coefficients which stand for the odds ratio of probability of success to the probability of failure, SE = standard error of the estimate, Wald statistics = Wald statistics denotes relationship between dependent and independent variables; df = degree of freedom, Sig. = significance or P values, Exp(β) = odds ratio (probability of success over probability of failure)

* Statistically significant at P < 0.05 level

#### Age

A number of adaptation strategies were observed to decrease as the individuals in the household approaches old age. The negative beta coefficient (β) indicated that as the age of the respondent increases, there is likelihood of decrease in the number of adaptation strategies. Findings from this study are inconsistency from other literatures. Dolisca et al. (2006) explained that, age relates significantly to farmer’s decisions to adopt new technologies, thus affects adaptation to climate change. Similarly according to Tazeze et al. (2012), as age of the household head increases, the person is expected to acquire more experience in weather forecasting that helps increasing the likelihood of practicing different adaptation strategies to climate change. The differences observed could be due to difference in life expectancy between Tanzania compared to Ethiopia and Haiti.

#### Household size

It was revealed that, household size had positive beta coefficient (β) suggesting significant influence (P ≥ 0.05) on the number of adaptation strategies a household can contain. That implied a unit change in household size increases likelihood of increasing the number of adaptation strategies (Table 10). It is anticipated that, a household with large number of individuals increase adaptive capacity to climate change (Nhemachena and Hassan 2008; Aymone 2009). A study by Giliba et al. (2010) in Babati district and Njana et al. (2013) at Urumwa Forest Reserve in Tabora region showed that household sizes facilitated the contribution of the livelihoods of local people adjacent to forests resources. Similarly, Gbetibouo (2009) in South Africa revealed that a large household is more willing to choose the adaptation options that are labour intensive such as soil conservation techniques, developing irrigation schemes and chemical treatments. Household size determines per capita livelihood diversification hence contribution to people’s adaptive capacity (Ellis 2000; Gbetibouo 2009).

#### Residence period

The residence period the head of household lived in the study area had significant positive beta coefficient (β) (P ≥ 0.05), suggesting that it influences adoption of developed adaptation strategies. This implied that, a unit change in resident period increases likelihood of increasing the number of adaptation strategies (Table 10). However, there were no significant differences (P ≥ 0.05) in residence period when more than three adaptation strategies were involved. It is speculated that more adaptation strategies were probably developed by people who lived in the study area for a long time because of increased experience, knowledge and gained information on adverse climate change effects. According to Ellis (2000) and Nhemachena and Hassan (2008) people living for a long period in a certain area are able to develop large number of adaptation strategies.

#### Land ownership

Land ownership is about securing right to long term access to land and their benefits (Lutz et al. 1994). Results showed that land ownership had positive beta coefficient (β) suggesting that it influences the number of adaptation strategies especially when people decided to have more than three options (Table 10). The variable was not significant (P ≥ 0.05) when people opted to have two to three adaptation strategies. This could probably be associated with the security on land ownership. Secured ownership enables people to have various adaptation strategies developed on the particular land. Farmers who own land are more likely to invest in various adaptation options, including crop and livestock management practices and water conservation. Studies show that land ownership encourages the adoption of various technologies linked to land including irrigation, drainage, tree planting and crop diversification (Lutz et al. 1994; Shultz et al. 1997; Nhemachena and Hassan 2008).

#### Household income

Table 10 indicates that household income had positive beta coefficient (β) suggesting that the variable has influence on adoption of adaptation strategies forest adjacent households developed. This indicates that, a unit change in this variable increases likelihood of increasing the number of adaptation strategies, which was statistically significant (P ≥ 0.05). It is assumed that households with higher income and greater assets are in better position to adopt new farming technologies than those with less. Findings in this study are similar to that of Deressa et al. (2009) and Tazeze et al. (2012) who found that household income had significant impact on increasing adaptation strategies which include, use of different crop varieties, irrigation technologies, early cultivation and changing planting dates.

#### Education level

Results have also revealed that education of the respondent had positive beta coefficient (β) suggesting that it influences the number of adaptation strategies, though was not statistically significant (P ≥ 0.05). Studies by Adesina and Forson (1995) and Daberkow and McBride (2003) showed that educated and experienced farmers have more knowledge and information about climate of a particular area, thus enabling individuals increase the probability of adopting new technologies when adversely affected. Similarly, Tazeze et al. (2012) found that literate farmers are more likely to respond to climate change by making best adaptation options based on preferences and influences of individual decision making.

#### Forest access

Forest access is important when it comes to the issue of livelihood contribution to forest adjacent households. The rules and regulations put on access to the forest resources often influence the livelihoods of the forest adjacent households. Results showed that forest access rules had negative beta coefficient (β) in the study area suggesting its less influence on the number of adaptation strategies a household can contain (Table 10). As the beta coefficient of this variable was negative, it implied a unit change decreases the likelihood of increasing the number of adaptation strategies. In the study area, forest adjacent households declared that, recently collection of forest products from IFR [(now part of Kilombero Nature Reserve (KNR)] and grassland is not granted, and thus access has been illegal. Free access was on village woodlands and on farms. However discussing with the forest officer in charge, it was revealed that currently there is informal arrangement that allow forest adjacent households to enter into the forests to collect some few non-destructive NTFPs like dried firewood, mushrooms, fruits and wild vegetables. This type of informal arrangement could not be sustainable as it depended on the willingness of the conservator in charge at that time. One of the key informants at Mpofu Village reported that:

- …we are currently informally allowed to collect some products like dried firewood, mushrooms, fruits, and wild vegetable from IFR. However, due to changes in management regime made to the forest, we might not be able to access the forest in future; the government should consider our livelihoods

It was further noted that law enforcement was mainly on harvesting timber, building poles and hunting of wild games in the reserve. However, not all of the forest adjacent households were well informed about the prevailing legal status. This calls for the need for raising awareness. The findings in this study are in line with those by Mombo et al. (2011) who indicated that, majority of the local people were not aware whether there was specific law prohibiting them from performing specific activities in the Kilombero Valley Floodplain Ramsar Site (KVFRS).

## Conclusions and recommendations

### Conclusions

The study concludes that changes in climatic conditions are impacting the livelihood security of households adjacent to forest resources in Kilombero District. These changes, in terms of increasing dry spells, floods, heavy rains, high temperature, pest and diseases are associated with climate change. The findings contribute to empirical evidence that people in humid areas are also experiencing adverse impact of climate change on their livelihoods similar to what is already known from arid and semi-arid areas of Tanzania. This understanding is particularly important because research and policy have paid less attention to the vulnerability of rural populations in humid areas like Kilombero. In responding to adverse effects of climate change, forest adjacent households have developed local adaptation strategies which are farm and non-farm including crop diversification, changing cropping calendar, adopting modern farming techniques and livestock keeping. Reliance on NTFPs to cope with flood effects, pests and diseases cannot be overemphasized. These findings elaborated understanding of how NTFPs contribute to livelihood capital assets and how they play in management of environmental stresses by different households. NTFPs use in the context of local adaptation strategies must therefore be understood as fulfilling multiple roles and functions that complement and strengthen other non-forest based strategies rather than contributing only to subsistence or single types of capital. To attain good number of adaptation strategies/options, some socio-economic variables including household size, residence period the respondent lived in the study area, land ownership and household income were significantly important in the study area.

### Recommendations

There is a need for actual implementation of climate change local policies and strategies at household levels. This is because livelihoods of people from different agro ecological zones of Tanzania are insecure in the face of adverse effects of climate change. Particularly, the study calls for interventions on how to manage both dry spells and floods as they both appear and have adverse effects on the livelihoods of the forest adjacent households. Attention needs to be directed at enhancing adaptive capacity among populations in a wide range of geographical locations particularly to the forest adjacent households. Actual implementation of climate change local policies and strategies is pertinent in order to enhance adaptive capacity at household level. It is suggested that, formal arrangement be established in order to enable forest adjacent households’ access the forest for NTFPs collection.
